# Correlation Between Sociocultural and Economic Factors in Pediatric Patients' Families and Emergence Delirium

**DOI:** 10.7759/cureus.46229

**Published:** 2023-09-29

**Authors:** Tuba K Yoldas, Cengiz Sahutoglu, Ozgecan Kaynarca, Canan Bor

**Affiliations:** 1 Anesthesiology and Reanimation, Ege University School of Medicine, İzmir, TUR

**Keywords:** emergence delirium, educational status, economic status, pediatrics, ophthalmologic surgical procedure

## Abstract

Background: Postoperative delirium is a commonly encountered condition that can arise from multiple factors, and its occurrence varies based on the type of surgery in pediatric patients. This study aimed to investigate the occurrence of delirium during the recovery from anesthesia in children undergoing eye surgery and its association with the sociocultural and economic status of their families.

Methods: This prospective observational study included children aged 2-12 years who underwent eye surgeries. Demographic data, socioeconomic and educational status of parents, parental separation and cooperation scores, Cravero agitation score, and face, legs, activity, cry, and consolability (FLACC) score (at zero, five, 15, and 30 minutes in the postoperative period) were recorded. Patients who scored 5 on the Cravero agitation scale for at least five minutes were considered to have postoperative delirium. The STROBE checklist was followed for reporting.

Results: A total of 104 patients were included in the study, of which 65 were male. The mean age of the patients was 6.5±2.9 years, and 42 patients (40.4%) belonged to the preschool age group. The incidence of delirium was found to be 51.9%. Delirium was found to be associated with postoperative pain (p=0.003), age (p=0.001), preoperative anxiety (not cooperative examination score (p=0.047), poor separation score (p=0.006)), presence of a surgical history (p=0.012), and cataract surgery (p=0.007). No evidence was found to demonstrate a link between sociocultural and economic conditions and the development of delirium.

Conclusions: This study identified several factors that influenced the occurrence of delirium, including postoperative pain (FLACC≥4), younger age (<6 years), cataract surgery, presence of surgical history, examination score (score 3, not cooperative), and separation score (scores 3-4, poor).

## Introduction

Postoperative delirium is a prevalent complication that occurs after anesthesia, characterized by acute cerebral dysfunction and fluctuating attention-cognitive impairment. The incidence of postoperative delirium varies among different patient populations. In critical pediatric intensive care units, delirium prevalence ranges from 12% to 28% [1]. In children undergoing major surgeries, such as cardiac surgery and brain surgery, the incidence of postoperative delirium is reported to be approximately 66%; in a group of patients undergoing cardiothoracic pediatric surgery, this rate is reported to be between 49% and 57% [2,3].

Several risk factors contribute to the development of postoperative delirium, including preschool age, presence of pain, type of surgery, anesthetic agents used, duration of anesthesia, anesthesia techniques, rapid recovery from inhalation anesthesia, noisy environment, and postoperative anxiety [4,5]. Postoperative anxiety is a significant risk factor for delirium and is more frequently observed in children compared to adults [6]. Notably, otolaryngology and ophthalmology procedures are commonly associated with postoperative delirium [4]. For example, ocular bandages used after ophthalmic surgery can obstruct vision and lead to postoperative agitation [7]. Postoperative delirium contributes to delayed discharge from the post-anesthetic care unit (PACU), reduced family satisfaction, and increased hospital costs [8].

In this study conducted at a tertiary-level hospital, we aimed to investigate the relationship between the incidence of postoperative delirium and the sociocultural and economic status of families in pediatric patients undergoing strabismus, cataract, and glaucoma surgeries.

## Materials and methods

From June 2019 to May 2021, we conducted a prospective observational study involving 104 American Society of Anesthesiologists (ASA) physical status class I-II pediatric patients, aged 2-12 years who were scheduled for cataract, glaucoma, and strabismus surgeries. The study was approved by the Clinical Research Ethics Committee (protocol number: 19-5.2T/59, date: 29/05/2019), and written consent was obtained from the parents of the patients. All procedures were performed in accordance with the Helsinki Declaration-2013. Six patients were excluded from the study due to missing information in their medical records. Additionally, patients with psychiatric-neurological disorders, central nervous system diseases, and hearing impairment were excluded from the study.

Demographic data, including age, weight, height, educational status, and ASA score, were recorded for each patient. The preoperative anesthetic physical examination was also documented. Furthermore, a questionnaire was administered to collect information on the socioeconomic status indicators of the patients' families, including place of residence, monthly income, hometown, nuclear/extended family, daycare arrangements for the child, and the age and educational status of the mother and father.

To facilitate anesthesia induction, age-appropriate games were utilized, and premedication was not administered prior to transporting the patients to the operating room. Upon admission to the operating room, separation scores indicating the patients' ability to separate from their family (rated on a 4-point scale: 1 = excellent, 2 = good, 3 = fair, and 4 = poor), and cooperation scores reflecting the patient's compliance with mask usage during inhalation anesthesia (rated on a 3-point scale: 1 = easy, 2 = slightly resistant, and 3 = markedly resistant) were evaluated and recorded.

Anesthesia induction was initiated using 8% sevoflurane and an 80% oxygen-air mixture delivered via a face mask. Standard monitoring techniques, including electrocardiography, noninvasive blood pressure measurement, pulse oximetry, and capnography, were employed for all patients. After induction, intravenous administration (IV) of 0.01 mg/kg atropine, 0.6 mg/kg rocuronium (only in orotracheally intubated patients), 0.5 mcg/kg remifentanil, and 0.5 mg/kg dexamethasone was added. Airway management was tailored to the specific needs of each surgical procedure. Supraglottic airway devices were employed for cataract and glaucoma patients with anticipated operating times of under one hour, while endotracheal tubes were utilized for glaucoma patients with strabismus and expected operating times exceeding one hour. Anesthesia was maintained with 3% sevoflurane, 50% oxygen, and 50% air mixture, and end-tidal CO₂ levels were maintained at 30-35 mmHg. Since a neuromuscular blocker was not required in all patients, the train-of-four (TOF) monitor was not used as a standard in the study. Sugammadex (2 mg/kg) was used for the reversal of neuromuscular blockade.

For postoperative analgesia, all patients received intravenous administration of 10 mg/kg paracetamol. The operated eye was covered with a pad and a transparent eye shield. The duration of anesthesia and surgery for each patient was recorded. In the PACU, at the 15th minute, one of the parents was allowed to visit the patient. The face, legs, activity, cry, and consolability (FLACC) score was utilized to evaluate the postoperative pain status at zero, five, 15, and 30 minutes. A FLACC score of ≥ 4 was established as an indicator of pain [9], and fentanyl 0.5 microgram/kg was added as a rescue analgesic to the patients. Postoperative agitation and delirium were assessed at zero, five, 15, and 30 minutes using the Cravero emergence agitation score [10] (Table 1). Patients who demonstrated Cravero agitation scale score of 5 for a minimum of five minutes were categorized as having postoperative delirium, and they received a slow intravenous administration of dexmetomidine at a dosage of 0.5 micrograms per kilogram.

**Table 1:** Cravero emergence agitation scale (scoring system for emergence delirium). A score of 5 was considered delirium [10].

Behavior	Score
Obtunded with no response to stimulation	1
Asleep but responsive to movement or stimulation	2
Awake and responsive	3
Crying (for >3 min)	4
Thrashing behavior that requires restraint	5

Statistical analyses

The incidence of delirium in an observational study conducted during ophthalmic surgery was found to be 46.9% [11]. To determine the appropriate sample size with a significance level of 5% and a power level of 80%, we calculated a sample size of 94 patients. To account for potential patient exclusions, we increased the sample size by 10%, resulting in a total of 104 patients.

Data collected from the study were analyzed using Statistical Product and Service Solutions (SPSS) (version 24; IBM SPSS Statistics for Windows, Armonk, NY). Descriptive statistics were employed to summarize the distribution of responses for independent variables. Categorical variables were presented as frequencies and percentages, while numerical variables were expressed as means, standard deviations, and medians. The chi-square test was used for paired and multiple comparisons of categorical variables, while the independent t-test and one-way ANOVA were employed for quantitative variables. The Tukey test was performed for posthoc comparisons of quantitative data among more than two groups. Logistic regression analysis (method = backward stepwise (likelihood ratio)) was used to identify risk factors for delirium. Statistical significance was determined at a p-value of p < 0.05, and the results were interpreted using a corresponding 95% confidence interval. The STROBE checklist was followed for reporting.

## Results

A total of 104 patients aged 2-12 years were included in the study, with 65 (62.5%) being male. The mean age of the patients was 6.5±2.8 years, and the majority (85.6%) were classified as ASA class I, and 47.1% were preschoolers. Among the surgeries performed, 38.4% were for cataracts (n=40), 17.3% for glaucoma (n=18), and 44.2% for strabismus (n=46). Laryngeal mask airway (LMA) insertion was used for anesthesia in 65 (62.5%) patients, while 39 (37.5%) underwent orotracheal intubation. The median durations for surgery, anesthesia, and extubation were 42.5 minutes (range: 15-120), 50 minutes (range: 25-135), and five minutes (range: 2-12), respectively. The age, weight, and height of patients in the delirium group were lower. Delirium occurred more frequently in patients under six years of age and in the preschool-age group. Additionally, it was more prevalent in patients who received LMA anesthesia and underwent cataract surgery (Table 2).

**Table 2:** Patients’ demographics and peroperative data. The data were presented as either mean ± standard deviation, median (minimum-maximum), or number of patients (percent). * p<0.05 is statistically significant. Abbreviations: kg: kilogram, cm: centimeter, ASA: The American Society of Anesthesiologists physical status classification system, LMA: laryngeal mask airway, ETT: endotracheal tube

		No Delirium (n=50)	Delirium (n=54)	p
Age (years)		7.5±2.7	5.6±2.6	<0.001*
	(<6 year)	12 (33.3)	24 (66.7)	0.029*
Gender (male)		27 (54)	38 (70.4)	0.085
Height (cm)		126±20	113±16	<0.001*
Weight (kg)		25 (12-68)	20 (10-59)	0.001*
ASA score	I	40 (80)	49 (90.7)	0.119
	II	10 (20)	5 (9.3)	
Education level	Preschool	14 (28)	28 (51.9)	0.007*
	Primary	26 (52)	24 (44.4)	
	Secondary	10 (20)	2 (3.7)	
Surgery history	(Yes)	24 (48)	35 (64.8)	0.084
Type of surgery	Cataract	11 (22)	29 (53.7)	0.003*
	Glaucoma	10 (20)	8 (14.8)	
	Strabismus	29 (58)	17 (31.5)	
Duration of surgery (minute)		40 (20-115)	45 (15-120)	0.090
Type of anesthesia	LMA	25 (50)	40 (74.1)	0.011*
	ETT	25 (50)	14 (25.9)	
Duration of anesthesia (minute)		45 (20-120)	40 (15-115)	0.127
	≥ 60 min	23 (46)	18 (33.3)	0.187
Extubation time (minute)		5 (2-12)	5 (2-8)	0.441

The overall incidence of postoperative delirium was 51.9%. At specific time intervals, we observed varying rates of patients experiencing delirium, as indicated by the Cravero agitation scale 5: at zero minutes, 22 patients (21.2%); at five minutes, 17 patients (16.3%); at 15 minutes, 16 patients (15.4%); and at 30 minutes, six patients (5.8%). During the assessment using the FLACC pain score, a noteworthy 73.1% of patients in the postoperative period reported high pain scores. To address this pain, fentanyl was administered as analgesia. Parental separation scores (scores 3-4) and cooperation scores (scores 2-3) had higher rates of the delirium group (p<0.05) (Table 3).

**Table 3:** Assessments of the patients’ examination suitability, separation scores, cooperation scores, Cravero agitation scores, and pain scores. The data were presented as number of patients (percent), and median (minimum-maximum). * p<0.05 is statistically significant. Abbreviations: FLACC: face, legs, activity, cry, and consolability

		No Delirium (n=50)	Delirium (n=54)	p
Examination suitability	Cooperative (Score 1-2)	27 (54)	25 (46.3)	0.432
	Not cooperative (Score 3)	23 (46)	29 (53.7)	
Separation Score	Score 1-2 (Good)	32 (64)	24 (44.4)	0.046*
	Score 3-4 (Poor)	18 (36)	30 (55.6)	
Cooperation Score	Score 1	25 (50)	14 (25.9)	0.011*
	Score 2-3	25 (50)	40 (74.1)	
Cravero Agitation Score	0. min	2 (1-5)	2 (1-5)	0.168
	5. min	2 (1-5)	4 (1-5)	<0.001*
	15. min	3 (1-4)	4 (4-5)	<0.001*
	30. min	3 (1-4)	4 (2-5)	<0.001*
Pain (FLACC ≥ 4)	0. min	18 (36)	30 (55.6)	0.046*
	5. min	15 (30)	27 (50)	0.038*
	15. min	12 (24)	37 (68.5)	<0.001*
	30. min	7 (14)	37 (68.5)	<0.001*
Postoperative pain		27 (54)	49 (90.7)	<0.001*

In terms of sociocultural and economic factors, it was observed that the majority of the parents of the patients fell within the 30-40 age bracket and had completed primary school education. Children whose fathers had completed primary school education showed a higher incidence of delirium (p=0.014). In terms of monthly income in 2017, 63.5% of the families had an income below 1,000 dollars (low), while 13.5% had an income above 2,000 dollars (high). The sociocultural and economic data of the families are presented in Table 4.

**Table 4:** Sociocultural and economic data of the patients’ families. The data were presented as the number of patients (percent).

		No Delirium (n=50)	Delirium (n=54)	p
Place of residence	Village	5 (10)	4 (7.4)	0.874
	Town	17 (34)	20 (37)	
	City	28 (56)	30 (55.6)	
Mother’s age	20-30	7 (14)	7 (13)	0.755
	31-40	32 (64)	38 (70.4)	
	>40	11 (22)	9 (16.7)	
Mother’s educational status	No/Literate	2 (4)	10 (18.5)	0.113
	Primary	19 (38)	23 (42.6)	
	Secondary	8 (16)	8 (14.8)	
	High School	15 (30)	9 (16.7)	
	University	6 (12)	4 (7.4)	
Father’s age	20-30	3 (6)	1 (1.9)	0.479
	30-40	28 (56)	34 (63)	
	>40	19 (38)	19 (35.2)	
Father’s educational status	No/Literate	1 (2)	8 (14.8)	0.014
	Primary	16 (32)	24 (44.4)	
	Secondary	5 (10)	7 (13)	
	High School	19 (38)	5 (9.3)	
	University	9 (18)	10 (18.5)	
Family income level	Low	28 (56)	38 (70.4)	0.115
	Middle	16 (32)	8 (14.8)	
	High	6 (12)	8 (14.8)	
Family size	Nuclear Family	42 (84)	51 (94.4)	0.084
	Extended Family	8 (16)	3 (5.6)	
Child’s daycare	Mother/Family Elder	28 (56)	35 (64.8)	0.358
	Pre-School/School	22 (44)	19 (35.2)	

Logistic regression analysis revealed that postoperative pain (FLACC≥4) (p=0.003), younger age (p=0.001), not cooperative examination suitability (p=0.047), poor separation score (p=0.006), presence of a surgical history (p=0.022), and cataract surgery (p=0.007) were identified as independent risk factors for postoperative delirium (Table 5).

**Table 5:** Risk factors for pediatric delirium (logistic regression analysis). Abbreviations: FLACC: face, legs, activity, cry, and consolability, B: standardized coefficients beta, OR: odds ratio, CI: confidence interval

	B	p	OR	95% CI for OR
Pain (FLACC ≥ 4)	2.714	0.003	15.095	2.546-89.516
Age	-0.466	0.001	0.628	0.472-0.835
Examination suitability (Not cooperative)	2.537	0.047	12.636	1.034-154.422
Separation Score (Score 3-4. Poor)	3.516	0.006	33.637	2.761-409.727
Surgery history (Yes)	1.959	0.012	7.091	1.541-32.641
Type of surgery (Glaucoma)		0.022		
Cataract	2.499	0.007	12.166	2.035-73.800
Strabismus	1.335	0.151	3.802	0.615-23.483
Father’s age (>40 years)		0.063		
20-30 years	-3.114	0.060	0.044	0.002-1.134
31-40 years	0.629	0.376	1.875	0.467-7.531
Father’s educational status (University)		0.129		
No/Literate, Primary	-1.334	0.395	0.263	0.012-5.683
Secondary/High School	-2.320	0.065	0.098	0.008-1.157
Mother’s educational status (University)		0.143		
No/Literate, Primary	3.273	0.071	26.381	0.757-919.204
Secondary/High School	2.886	0.054	17.922	0.948-338.685
Constant	-7.573	0.022	0.001	

## Discussion

In our study, we found a postoperative delirium rate of 51.9% in pediatric patients aged 2-12 years undergoing ophthalmic surgery. Our findings highlight the influence of various factors on the occurrence of delirium, including postoperative pain, younger age, preoperative anxiety (not cooperative examination score, poor separation score), presence of surgical history, and cataract surgery. No evidence was found to demonstrate a link between sociocultural and economic conditions and the development of delirium.

The incidence of delirium can vary depending on the type of surgery. In pediatric patients undergoing surgery, delirium rates can reach up to 66% [2,12]. Previous studies have indicated higher rates of recovery agitation following ophthalmic and ear, nose, and throat surgeries, suggesting the significance of these procedures as determinants of delirium [13]. Eshetie et al. [13] reported a higher incidence of delirium in ophthalmic and ear-nose-throat surgeries (56.2%) compared to other surgical procedures (32.7%). Ophthalmic surgeries, in particular, have a higher incidence of postoperative delirium due to changes in vision following the operation compared to other pediatric surgeries [14]. Consistent with the literature, our study found a high incidence (51.9%) of delirium following eye operations.

Various methods are available for preventing postoperative agitation and delirium. One approach involves covering the eye for three hours a day prior to eye operations, allowing the patient to gradually adapt to the vision changes after surgery, which has been shown to reduce agitation [5]. Dong et al. [15] conducted a study comparing the effects of 30-minute and 60-minute eye-covering on preschool children before eye surgery, in comparison to a control group. Their research assessed preoperative anxiety scores, postoperative delirium scores, and the incidence of delirium cases in both the 30-minute and 60-minute closure groups, as well as the control group. The findings revealed that both the 30-minute and 60-minute eye-covering groups exhibited lower preoperative anxiety scores, lower postoperative delirium scores, and a reduced incidence of delirium when compared to the control group. Interestingly, pain scores were found to be similar across all three groups. Furthermore, it is worth noting that delirium was significantly higher in the control group, with a prevalence of 77.6%, whereas the 30-minute and 60-minute closure groups exhibited lower rates of delirium, at 26.3% and 34.7%, respectively [15]. In our study, we did not employ this procedure before the operation, so a high incidence of delirium may have been observed.

Age group is recognized as a factor that can influence postoperative delirium [7,12,16]. Many studies have highlighted a higher occurrence of delirium in preschoolers, particularly in patients aged 2-5 [7,13,16]. Similar to the literature, in our study, patients, being under six years of age, were a risk factor for delirium. The risk decreases with increasing age. While the precise mechanism remains unclear, it is evident that individuals within this age group exhibit a heightened sensitivity to both immobilization and disruptions in their sleep patterns following surgery [17].

Regarding the type of airway device used, Eshetie et al. [13] reported delirium incidence as 46.7% in patients with an LMA and 52.9% in those with an endotracheal tube. Although our study did not determine the effect of anesthesia duration on delirium incidence, some studies have suggested that longer anesthesia administration may increase the risk of postoperative agitation. However, conflicting findings have been reported by other studies, which found no significant difference in anesthesia duration between patients with and without delirium [18,19]. In our study, we observed no effect of anesthesia duration standardized anesthesia induction, and maintenance protocols were employed for all patients. Nevertheless, because we employed LMA during cataract surgery, and cataract surgery itself represents a risk factor for delirium, we observed elevated delirium rates associated with the use of LMA.

Children who undergo surgery under general anesthesia may have experienced significant anxiety prior to the procedure, potentially resulting in non-cooperation. They might witness inappropriate behavior during preoperative evaluation, feel distressed about being separated from their parents, and find the application of the anesthesia mask unsettling. If this anxiety is not addressed, it can lead to challenges in inducing anesthesia, amplified postoperative pain, a greater requirement for additional pain relief, and an increased risk of delirium [20,21].

Estesia et al. [13] and Saringcarinkul et al. [16] highlighted a higher prevalence of postoperative delirium in children with higher family separation scores. Dagli et al. [22] found higher scores in preoperatively anxious children on the modified Yale preoperative anxiety scale, separation score, cooperative score, and induction compliance checklist score. In our study, the delirium group had significantly higher separation and cooperative scores. However, only the separation score and examination suitability were determined to be independent risk factors. These results show that preoperative anxiety is effective on delirium and the anxiety should be prevented.

Dagli et al. [22] reported a higher incidence of delirium in the low- and middle-income groups. However, the educational status and family size of the parents did not influence delirium. Wu et al. [23] identified a greater occurrence of intensive-care delirium among low-income patients. However, their multivariate analyses did not reveal a statistically significant correlation between low income, low education levels, and delirium.

In our study, no relationship was found between the age and education level of the parents, family income, family size, and the presence of delirium. Since most of our patients were from low- and middle-income groups, no significant results may have been found. Parents residing in rural areas with limited education and lower income tend to experience heightened levels of anxiety, which can be transferred to their children [24].

Postoperative pain is recognized as a contributing factor to postoperative agitation and is sometimes mistaken for delirium. It is not easy to distinguish pain from agitation, especially in preschool pediatric patients. Sethi et al. [25] reported pain-induced delirium in cataract surgery, while Eshetie et al. [13] found no relationship between pain and delirium. In our study, we found high FLACC scores in patients experiencing agitation and delirium, indicating the presence of pain. Once pain control was achieved, the incidence of delirium decreased in patients. Therefore, effective pain management is crucial in the postoperative period and can significantly reduce agitation in patients.

Our study has several limitations: First, the patients were not premedicated before the operation. This may have resulted in an increased incidence of delirium. Second, the specific agents used and their dosages were not documented. Since it was an observational study, it focused only on the incidence of delirium and its risk factors. Finally, the eye closure exercise could not be applied to the children in the preoperative period, which may have contributed to higher delirium rates as the patient's eyes were closed.

## Conclusions

In conclusion, our study highlights the persistent occurrence of delirium in ophthalmic surgery. We identified significant correlations between postoperative delirium and factors such as younger age, postoperative pain, preoperative anxiety (not cooperative examination, poor separation score), a history of prior surgery, and cataract surgery. Importantly, our findings do not support a connection between parents' sociocultural and economic conditions and the development of delirium. To mitigate the incidence of postoperative delirium, it is crucial to provide comprehensive information to both the family and the child prior to the operation, supported by visual materials. Additionally, taking proactive measures to address the sociocultural status of the families and effectively managing postoperative pain will contribute to reducing the occurrence of delirium.
