# Correction: Correlation Between Sociocultural and Economic Factors in Pediatric Patients' Families and Emergence Delirium

**DOI:** 10.7759/cureus.c139

**Published:** 2023-10-13

**Authors:** Tuba K Yoldas, Cengiz Sahutoglu, Ozgecan Kaynarca, Canan Bor

**Affiliations:** 1 Anesthesiology and Reanimation, Ege University School of Medicine, İzmir, TUR

This article has been corrected to remove asterisks denoting statistical significance from three p-values in Table 2: 0.085, 0.084 and 0.090. Additionally Table 4 and 5 were accidentally transposed and have been switched to the correct order. The tables themselves were not altered. The authors and journal regret that these errors were not identified and corrected prior to publication.

